# Validity of Questionnaire and Representativeness of Objective Methods for Measurements of Mechanical Exposures in Construction and Health Care Work

**DOI:** 10.1371/journal.pone.0162881

**Published:** 2016-09-20

**Authors:** Markus Koch, Lars-Kristian Lunde, Tonje Gjulem, Stein Knardahl, Kaj Bo Veiersted

**Affiliations:** 1 Department of Work Psychology and Physiology, National Institute of Occupational Health, Oslo, Norway; 2 Oslo University Hospital, Oslo, Norway; Columbia University, UNITED STATES

## Abstract

**Objectives:**

To determine the criterion validity of a questionnaire on physical exposures compared to objective measurements at construction and health care sites and to examine exposure variation over several working days.

**Methods:**

Five hundred ninety-four construction and health care workers answered a baseline questionnaire. The daily activities (standing, moving, sitting, number of steps), postures (inclination of the arm and the trunk), and relative heart rate of 125 participants were recorded continuously over 3–4 working days. At the end of the first measurement day, the participants answered a second questionnaire (workday questionnaire).

**Results:**

All objective activity measurements had significant correlations to their respective questions. Among health care workers, there were no correlations between postures and relative heart rate and the baseline questionnaire. The questionnaires overestimated the exposure durations. The highest explained variance in the adjusted models with self-reported variables were found for objectively measured sitting (R^2^ = 0.559) and arm inclination > 60° (R^2^ = 0.420). Objective measurements over several days showed a higher reliability compared to single day measurements.

**Conclusions:**

Questionnaires cannot provide an accurate description of mechanical exposures. Objective measurements over several days are recommended in occupations with varying tasks.

## Introduction

Musculoskeletal disorders (MSD) are the most prevalent cause of sickness absence and early retirement [1,2]. There is a high prevalence of MSD in occupations with high physical demands [2]. Mechanical exposures at work, such as repeated movements, heavy physical load [2], vibrations and awkward postures [3], and psychosocial exposures [4] are risk factors for work-related MSD [5]. Valid measures of mechanical exposures are pivotal in determining risk factors in efforts to reduce the occurrence of MSD. Mechanical exposures are characterized by the type of work and postures, movements, and exerted forces measured in terms of level, duration, and frequency [6,7]. The assessments may be based on self-reports, observational methods and direct measurements. The appropriate assessment method should be selected according to the study’s aims, the applicability and validity of these methods and economic aspects [8].

Self-reported assessments (e.g., questionnaires, diaries) of mechanical exposures at worksites have shown varying validity [9] and are often tested against observational methods with their own strengths and limitations [9–11]. For measuring physical activity, one review concluded that questionnaires have shown acceptable reliability [12], while Dyrstad and colleagues concluded that subjective measurements are inadequate [13]. For estimating movements and postures, data from questionnaires were found to have low correlations with data obtained with objective measurements by accelerometers [14]. Furthermore, self-reported measures seem to overestimate the duration of postural positions [15], and the errors were found to be dependent on the respondent’s occupation [16]. To obtain valid exposure measurements, objective measurements are recommended [12]. Several accelerometers attached to the participant’s body have been found to be a valid method for recording movements [17–19] and postures [20] over several days [17]. To measure work intensity or aerobic strain, the recording of heart rate (HR) is a valid method. A linear relationship was found between HR and oxygen consumption during exercise or work [21]. The RHR takes the individuals minimal and maximal HR into account and was chosen to describe the physical work load [22,23].

In a longitudinal study of people in occupations generally considered to have high physical demands–namely, construction and health care—we examined mechanical exposures using both methods: questionnaires at two different time points and objective measurements on several consecutive working days [24]. The aim of the present study was to determine the criterion validity [25] of the questionnaires at baseline and on the first day of the objective measurements, using valid objective methods as a comparative standard. Furthermore, we considered whether a one-day recording is representative of the exposures during a typical work week and aimed to determine the differences in exposures between consecutive working days.

## Methods

### Study population

In total, 1165 baseline questionnaires (construction workers: n = 580; health care workers: n = 585) were distributed to employees of four construction companies and two local health service distributors in the area of Oslo, Norway. Five hundred ninety-four participants (construction workers: n = 293, 50.3%; health care workers: n = 301, 51.8%) responded.

Of the responders, 178 people in construction work and 193 people in health care work were willing to participate in the technical measurements, and a sample of 125 people was examined (construction workers: n = 62; health care workers: n = 63) based on availability and work schedules. This sample was selected to provide a representative sample of the occupations examined in the study. An overview of the participants’ individual characteristics is presented in Table 1. The exclusion criteria for the study were inadequate skills in reading and writing Norwegian, known allergic reaction to plaster / tape / bandages, and a diagnosed cardiovascular or musculoskeletal disease that made it impossible for the subject to perform physical tests.

**Table 1:** Descriptive statistics of the samples.

		Technical measurements	
Participants		n = 125	
Age (years)		42.38 (SD 11.73)	
Height (cm)		173.64 (SD 9.64)	
Weight (kg)		76.85 (SD 13.64)	
		Gender	
		Male	Female
Construction work	Project manager / leader in construction work	5	0
	Carpenter	21	0
	Bricklayer	6	0
	Concrete worker	14	0
	Assistant worker	4	0
	Driver	0	0
	Foreman	7	0
	Engineer in construction work	2	1
Health care work	Leader health care work	1	5
	Nursing professional / nurse	0	15
	Registered nurse for the mentally handicapped	3	4
	Cook or kitchen helper	4	4
	Personal care worker in health services	5	17
	Cleaning worker	0	2
Other	Work with various tasks	2	2
	Other occupations	1	0
**Total**		**75**	**50**

### Ethical aspects

Prior to participation, all subjects were informed of the purpose and methods of the study and signed a written consent form. This study was conducted in accordance with the 1964 Helsinki Declaration and approved by the Regional Committee for Medical and Health Research Ethics in Norway (2014/138/REK sør-øst D).

### Study design

After answering the baseline questionnaire, the participants selected for the technical measurements underwent a physical examination by a nurse or a physician. If the participants were physically healthy, instruments for technical recordings were attached to the participant’s body at the beginning of a subsequent work day. The recordings were performed during work and leisure time on three to four consecutive work days, including at least two work days. At the end of the first day, the participants were asked to answer a second questionnaire (“workday questionnaire”). They were instructed to log the start and stop of their work and leisure periods or the removal of the sensors in a diary.

### Questionnaires

The present study included subjective reports of mechanical exposures [26], musculoskeletal and psychological complaints in the preceding four weeks [27], perceived exertion [28], seniority, weight, height, and smoking status from the baseline questionnaire. Mechanical exposures and musculoskeletal complaints were also measured a second time with the workday questionnaire.

#### Mechanical exposures

The questions regarding mechanical exposures had a common introduction: “How often in your daily work are you exposed to […]”. The participants were asked about the following exposures: work standing, work sitting, work with hands above shoulder height, work with forward-bent trunk, and work in which your breathing rate increases. The answer categories were “never”, “sometimes”, “approximately 25% of the time”, “approximately 50% of the time”, “approximately 75% of time”, and “all the time” and were re-coded on a scale from 0 (“never”) to 5 (“all the time”).

#### Physical demands

Exertion at work was measured with the question “How physically demanding is your work?” The question was answered on a 13-point scale ranging from “not at all” to “maximally demanding”.

#### Musculoskeletal and psychological complaints

Musculoskeletal (neck, shoulders, upper and lower back, hip, knees, ankles and feet, upper extremity, head) and psychological (fear, depression, fatigue) complaints were rated on a four-point scale for intensity (0 = not troublesome, 1 = a little troublesome, 2 = quite troublesome, 3 = seriously troublesome) and a four-point scale for duration (1 = 1–5 days, 2 = 6–10 days, 3 = 11–14 days, 4 = 15–28 days). For all complaints, a complaint severity score was calculated by multiplying the intensity score by the duration score (range 0–12). One musculoskeletal complaint severity index (MSI) and one psychological severity index (PSI) were calculated as the mean of all included complaint severity indexes [27].

#### Smoking status

Smoking status was measured on a four-point scale (1 = never, 2 = in the past, 3 = sometimes, 4 = every day).

### Instrumentation for technical measurements

To measure the acceleration, position and angle of various body segments of the participants, we used commercially available ActiGraph GT3X+ sensors (ActiGraph LLC, Pensacola, FL, United States). The ActiGraph GT3X+ is a tri-axial accelerometer that is small (46 x 33 x 15 mm), light (19 g) and waterproof. With a sampling frequency of 30 Hz, it allows data recording for up to 10 days continuously. Previous studies have found that the Actigraph GT3X+ sensors are valid for measuring the inclination of the upper arm and body during work tasks [20] and for detecting physical activity [18,19]. Four accelerometers were attached to the participant’s body as follows: dominant arm (3 cm below the deltoid muscle insertion), right upper leg (medially between the iliac crest and the upper crest of the patella), hip (top of iliac crest on the right side), and upper back (level T1-T2). The accelerometers were fixed to the skin, using double-sided tape (Fixomull, BSN medical, Hamburg, Germany) and covered with transparent film (Tegaderm, 3 M, Minnesota, United States).

To measure heart rate, an Actiheart monitor (Camntech, Cambridge, United Kingdom) was attached at the apex of the sternum and at the left intercostals at the level of the sixth and seventh costae [29]. Heart-rate monitors have been found to be valid and reliable for use both in the laboratory and in the field [30,31].

### Data and quality management

The raw data from the Actigraph sensors were stored on a personal computer using Actilife 6.11.5 software (Actigraph LLC, Pensacola, Florida, USA). The intensity and frequency of positions, various activities, and steps were calculated using the custom-made software Acti4 [18,20] based on the raw data and the participants’ diaries. Data were excluded when a sensor was not worn and when the work period was shorter than four hours or shorter than 75% of the mean average length of all working periods. The following variables were obtained: time spent standing, sitting and moving (movement in upright position, neither still or walking); the number of steps; the duration of arm inclination above 30°, 60°, 90°, 120° and 150° (IncArm); and trunk inclination along the sagittal plane greater than 20°, 30°, 60° and 90° (IncTrunk). These variables were normalized to one hour (e.g., steps per hour).

The relative heart rate (RHR) was calculated as follows [22]:

$${RHR}_{work}=\frac{\left(HRwork-HRmin\right)}{\left(HRmax-HRmin\right)}\;x\;100$$

HR_max_ was calculated for each participant using the formula 208–0.7 × age [32], and HR_min_ was based on a sex- and age-adjusted population [29]. Heart rate data were quality controlled visually and deleted if the beat error (a difference between two consecutive beats > 15, HR < 30, HR > 230) was higher than 50% for a work period. The data were calculated for each measurement day and averaged across all measurement days. Data processing was performed with Matlab R2013b (Math Works, Inc., Natick, Massachusetts, USA).

### Statistical analyses

The distributions of the variables were tested using the Kolmogorov-Smirnov test. The correlations between the questionnaire responses and the objectively measured data were calculated using Spearman’s rho, and the significance level was set as p = 0.005. The Spearman correlation coefficient was interpreted as follows: < 0.2: very low; 0.21–0.5: low; 0.51–0.7: moderate; 0.71–0.9: strong and > 0.9: very strong. The criterion validity of the exposure measurements was tested using linear regression analyses in two steps [33]. The objectively measured exposure variables were the dependent variables. The first step tested the corresponding subjective measurements for day 1, gender, height, weight, BMI, age, profession, work sector, MSI, PSI and smoking status separately as independent variables (unadjusted models). Those variables that exhibited associations with p-values < 0.1 were entered into a multiple linear regression for adjusted models. To determine the day to day reliability of objectively measured exposures, intraclass correlation coefficients (ICC) were calculated (single day measures: ICC 3, 1; average measures of 3 days: ICC 3, 3). To determine differences in objectively recorded mechanical exposures between consecutive working days, a Friedman one-way analysis of variance was used. The statistical data analyses were performed with IBM SPSS Statistics 22 (IBM Corporation, NY, United States).

## Results

The variables age, height, weight and objectively measured time spent standing and moving, trunk inclination > 20° and RHRmean were normal distributed. All other objectively measured variables were not normally distributed. There were no significant differences (p < 0.05) in age, height, weight, gender, MSI, PSI and smoking status between the questionnaire group at baseline (n = 594) and the group that underwent technical measurements (n = 125). Due to early removal of equipment or data not fulfilling quality criteria, some data were missing or had to be excluded. The total number of valid measurements from day one to day four were as follows: 125, 102, 72 and 27 (daily activities: 125, 101, 71, 27; Arm: 119, 96, 67, 27; Trunk: 121, 98, 66, 27; HR: 103, 83, 45, 13).

### Association between data from workday questionnaire responses and objective measurements of day one

**Fig 1:**
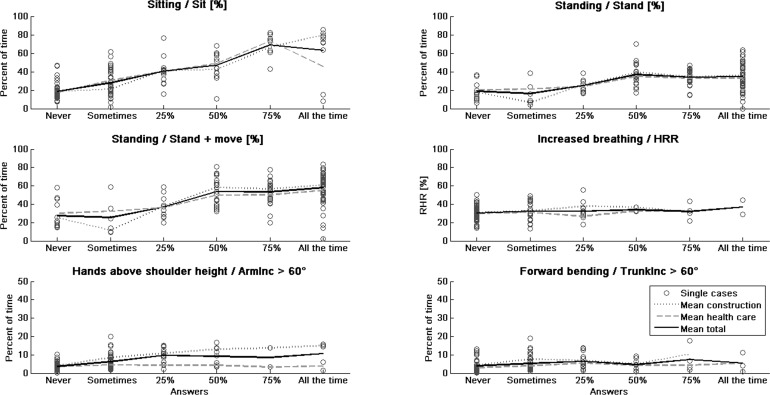
Categories of subjective vs. objective measures of exposures. The title of each subplot indicates the compared subjective and (/) objective variables. Single values (circles) of objective measures are plotted in the categories of the corresponding subjective measures. Mean values were calculated for each category for the total group (solid line), for construction workers (pointed line) and for health care workers (dashed line).

Fig 1 illustrates the amplitudes of the objective measurements compared with the responses to the corresponding subjective measurements.

#### Daily activities

Subjectively measured time spent standing showed moderate correlations with objectively measured time spent sitting and moving in all groups (p < 0.001). Furthermore, moderate correlations were found with objectively measured time spent standing and moving in the total group and the group of construction workers and with the number of steps in the group of construction workers (p < 0.001). Low correlations were found with objectively measured time spent standing and with the number of steps in the total group (p < 0.001) and with time spent standing and moving in the group of construction workers (p < 0.001). Moderate correlations were found in all groups for subjectively measured time spent sitting and objectively measured time spent sitting and moving (p < 0.001). Furthermore, moderate correlations were found between subjectively measured time spent sitting and objectively measured time spent standing and moving in the total group and the group of construction workers (p < 0.001) and with objectively measured number of steps in the total group and the group of health care workers (p < 0.001). Low correlations with objectively measured standing were found in all groups (p < 0.005), with time spent standing and moving in the group of health care workers (p < 0.001) and with the number of steps in the group of construction workers (p < 0.005).

#### Postures of the arm and the trunk

Objectively measured arm inclination > 60°, > 90°, and > 120° showed low correlations with the subjective measures of “work with hands above shoulder height” in the total group (p < 0.001). In the group of construction workers, there were moderate correlations between subjectively measured arm lifting and objectively measured arm inclination > 60° and > 90° (p < 0.001) and relatively low correlations with objectively measured arm inclination > 120° (p < 0.001). No significant correlations between subjectively and objectively measured arm inclination were found for the group of health care workers. For objectively measured trunk inclination > 60°, a low correlation was found with subjective measures in the total group (p < 0.005).

#### Physical exhaustion

No correlations were found between the self-reports of “How physically demanding was your work today?” and “How often were you exposed to increased breathing?” and RHR.

#### Quantitative relationships of subjective and objective measures

Regression analyses showed an explained variance of 18.9% for objectively measured standing in an adjusted model that included the variables subjectively measured time spent standing (β = 0.141, p < 0.001), age and profession (see Table 2). A variance of 34.6% for objectively measured time spent standing and moving could be explained by an adjusted model that included the variables subjectively measured standing (β = 0.285, p < 0.001), gender, age, profession and work sector. For objectively measured time spent sitting, 55.9% of the variance could be explained by an adjusted model that included the variables subjectively measured sitting (β = 0.498, p < 0.001), gender, age, profession and work sector (β = 10.199, p < 0.05; see Table 3).

**Table 2:** Correlations of objective measurements (Actigraph / Actiheart) and questionnaire responses (Spearman's rho).

		Workday questionnaire			Baseline questionnaire		
		-			-		
		Objective measurements on first measurement day			Objective measurements: mean of all days		
How often are you exposed to:	Objective measures:	Total	Construction work	Health care work	Total	Construction Work	Health care work
standing work?	Stand [%]	0.321**	0.292	0.311	0.526**	0.565**	0.501**
	Move [%]	0.563**	0.601**	0.502**	0.522**	0.483**	0.574**
	Stand + Move [%]	0.514**	0.546**	0.480**	0.506**	0.569**	0.409*
sitting work?	Sit [%]	0.686**	0.687**	0.538**	0.731**	0.732**	0.520**
work with hands above shoulder height?	IncArm >30° [%]	0.063	0.165	- 0.199	0.010	0.054	- 0.268
	IncArm >60° [%]	0.489**	0.732**	0.179	0.364**	0.514**	0.069
	IncArm >90° [%]	0.484**	0.646**	0.208	0.352**	0.458**	0.146
	IncArm >120° [%]	0.361**	0.454**	0.054	0.174	0.087	0.099
	IncArm >150° [%]	0.169	0.228	- 0.031	0.001	- 0.117	- 0.016
work with forward-bent trunk?	IncTrunk >20° [%]	0.076	0.089	0.063	- 0.068	- 0.037	- 0.069
	IncTrunk >30° [%]	0.162	0.206	0.155	0.084	0.205	0.004
	IncTrunk >60° [%]	0.267*	0.332	0.276	0.278*	0.361	0.198
	IncTrunk >90° [%]	0.228	0.318	0.271	0.147	0.209	0.112
increased breathing?	RHRmean [%]	0.108	0.152	- 0.029	0.123	0.089	0.040
How physically demanding is / was your work?							
	RHRmean [%]	0.225	0.401	- 0.033	0.280*	0.235	0.197

* p-value < 0.005

** p-value < 0.001.

**Table 3:** Unadjusted and adjusted regression analyses for objective und subjective measures.

**Standing**	** **	** **	** **	** **	** **	**Sitting**	** **	** **	** **
**Sub. measures:**	**Unadjusted**		**Adjusted**		**Sub. measures:**	**Unadjusted**		**Adjusted**	
	**β**	**p-value**	**β**	**p-value**		**β**	**p-value**	**β**	**p-value**
Standing	0.145	**0.000**	0.141	**0.000**	Sitting	0.500	**0.000**	0.498	**0.000**
Gender	-1.541	0.528	not included		Gender	7.166	0.065	-6.697	0.100
Height (cm)	-0.004	0.976	not included		Height (cm)	-0.106	0.597	not included	
Weight(kg)	0.028	0.755	not included		Weight(kg)	-0.017	0.906	not included	
BMI (kg/m^2^)	0.166	0.626	not included		BMI (kg/m^2^)	0.146	0.786	not included	
Age (years)	-0.189	0.066	-0.117	0.232	Age (years)	-0.189	0.066	0.186	0.117
Profession	-0.124	**0.041**	-0.111	0.051	Profession	0.188	0.054	-0.167	0.125
Work sector	-2.904	0.164	not included		Work sector	8.175	**0.014**	10.199	**0.033**
MSI	-0.014	0.982	not included		MSI	0.558	0.558	not included	
PSI	0.781	0.387	not included		PSI	-0.575	0.691	not included	
Smoking	0.387	0.711	not included		Smoking	-0.894	0.592	not included	
**Model summary: R**^**2**^ **adjusted = 0.189**					**Model summary: R**^**2**^ **adjusted = 0.559**				
**Standing + Moving **					**RHRmean**				
**Sub. measures: **	**Unadjusted**		**Adjusted**		**Sub. measures:**	**Unadjusted**		**Adjusted**	
	**β**	**p-value**	**β**	**p-value**		**β**	**p-value**	**β**	**p-value**
Standing	0.293	**0.000**	0.285	**0.000**	Physical demands	0.726	0.065	0.630	0.112
Gender	-4.694	0.174	not included		Increased breathing	0.915	0.206	Not included	
Height (cm)	0.080	0.652	not included		Gender	-3.108	0.073	-1.716	0.444
Weight(kg)	0.005	0.969	not included		Height	0.093	0.309	Not included	
BMI (kg/m2)	-0.122	0.799	not included		Weight	0.036	0.565	Not included	
Age (years)	-0.280	0.055	-0.146	0,246	BMI	0.029	0.901	Not included	
Profession	-0.170	**0.049**	-0.067	0,511	Age	-0.125	0.090	-0.088	0.245
Work sector	-6.024	**0.041**	-2.464	0,488	Profession	-0.058	0.152	Not included	
MSI	-0.223	0.805	not included		Work sector	-2.837	0.053	-1.323	0.491
PSI	1.015	0.429	not included		MSI	0.089	0.843	Not included	
Smoking	0.165	0.911	not included		PSI	-0.275	0.670	Not included	
**Model summary: R**^**2**^ **adjusted = 0.346**					Smoking	0.793	0.294	Not included	
					**Model summary: R**^**2**^ **adjusted = 0.084**				
**Arm inclination > 60**°					**Trunk inclination > 90**°				
**Sub. measures:**	**Unadjusted**		**Adjusted**		**Sub. measures:**	**Unadjusted**		**Adjusted**	
	**β**	**p-value**	**β**	**p-value**		**β**	**p-value**	**β**	**p-value**
Hands above shoulder height	0.080	**0.000**	0.063	**0.000**	Forward bended trunk	0.008	0.229	not included	
Gender	-3.529	**0.000**	-1.615	0.175	Gender	-0.688	**0.017**	0.334	0.493
Height (cm)	0.109	**0.010**	-0.099	0.093	Height (cm)	0.045	**0.002**	0.041	0.110
Weight(kg)	0.088	**0.003**	0.038	0.226	Weight(kg)	0.022	**0.042**	0.000	0.978
BMI (kg/m^2^)	0.171	0.137	not included		BMI (kg/m^2^)	0.009	0.826	not included	
Age (years)	-0.047	0.166	not included		Age (years)	-0.008	0.519	not included	
Profession	-0.062	**0.002**	0.033	0.221	Profession	-0.005	0.494	not included	
Work sector	-3.916	**0.000**	-3.918	**0.001**	Work sector	-0.714	**0.003**	-0.553	0.093
MSI	-0.281	0.194	not included		MSI	-0.097	0.216	not included	
PSI	-0.375	0.220	not included		PSI	-0.116	0.295	not included	
Smoking	0.414	0.246	not included		Smoking	0.152	0.223	not included	
**Model summary: R**^**2**^ **adjusted = 0.420**					**Model summary: R**^**2**^ **adjusted = 0.100**				

Regression analyses were calculated for all objectively measured arm inclination variables. The highest explained variance (42%) was calculated for arm inclination > 60° in an adjusted model that included the variables subjectively measured time with hands above shoulder height (β = 0.080, p < 0.001), gender, height, weight, profession and work sector (β = -3.918, p < 0.001).

For objectively measured trunk inclination, no significant regression model could be calculated that included subjective measurements of forward bending.

The regression analysis for the RHR showed no significant associations with the subjective measures “How physically demanding was your work today?” and “Increased breathing”, nor were the associations between RHR mean and gender, height, weight, BMI, age, profession and work sector significant. In total, the calculated beta values showed an overestimation of the times spent in various activities or postures. The overestimation was greater for time spent with arms above shoulder height or with a forward-bent trunk (see also Fig 1).

### Association of subjective reports (questionnaire at baseline) with the mean of objective measurements over several days

In the analysis of the mean values of objective measurements taken over several work days and the results of the baseline questionnaire, all groups showed moderate correlations for objectively and subjectively measured time spent standing (p < 0.001) and time spent sitting (p < 0.001).

Low correlations were found for objectively measured arm inclination > 60° and > 90° and subjectively measured hands above shoulder heights (p < 0.001), both for the total group and for the group of construction workers. Furthermore, objectively measured trunk inclination > 60° showed a low correlation with subjectively measured forward bending of the trunk in the total group and in the group of construction workers (p < 0.005).

A low correlation between RHR and the question “How physically demanding is your work?” was found only for the total group (0.280, p < 0.005).

### Day to day reliability of objective measurements

For all objectively measured variables, we found a higher ICC for the average measures over several working days than for the single day measures (see Table 4). Except for the number of steps in construction work, all of the average measures of daily activities showed a good or excellent reliability (range: 0.80–0.93). An arm inclination > 30° presented the highest ICC for all average measures of arm inclination (ICC 0.70, CI: 0.54–0.81) in the total group. Concerning arm inclination, construction workers had the highest ICC for average measures of arm inclination > 90° (ICC: 0.56, CI: 0.25–0.75), whereas health care workers showed the highest ICC for average measures of arm inclination > 30° (ICC: 0.84, CI: 0.66–0.93). Trunk inclination showed the highest degree of reliability in average measurements of trunk inclination > 20°. Health care workers showed higher ICCs for average measures of trunk inclination > 30° (ICC: 0.94, CI: 0.87–0.97), > 60° (ICC: 0.86, CI: 0.70–0.94) and > 90° (ICC: 0.82, CI: 0.62–0.92) than construction workers (ICC: 0.71, CI: 0.50–0.84; ICC: 0.37, CI: -0.06–0.65; ICC: 0.45, CI: 0.06–0.69, respectively). In all of the groups, the reliability for the average measures of RHRmean was good (range 0.84–0.89).

**Table 4:** Overview of intraclass correlation coefficients (95% confidence intervals) for objectively measured variables for the total group, construction and health care workers. For each variable, the ICC is presented for single day measures and for the average measures of 3 consecutive working days.

	Measures	Total	Construction work	Health care work
Time	Single	0.42 (0.27–0.57)	0.44 (0.25–0.62)	0.33 (0.08–0.59)
	Average	0.69 (0.53–0.80)	0.70 (0.50–0.83)	0.59 (0.20–0.81)
Sit [%]	Single	0.81 (0.73–0.88)	0.81 (0.70–0.88)	0.77 (0.59–0.89)
	Average	0.93 (0.89–0.95)	0.93 (0.88–0.96)	0.91 (0.81–0.96)
Stand [%]	Single	0.62 (0.49–0.74)	0.57 (0.40–0.72)	0.70 (0.48–0.85)
	Average	0.83 (0.75–0.89)	0.80 (0.67–0.89)	0.87 (0.74–0.94)
Move [%]	Single	0.68 (0.55–0.78)	0.68 (0.53–0.80)	0.63 (0.39–0.81)
	Average	0.86 (0.79–0.91)	0.86 (0.77–0.92)	0.84 (0.66–0.93)
Steps [Steps/h]	Single	0.59 (0.45–0.71)	0.50 (0.31–0.67)	0.69 (0.48–0.85)
	Average	0.81 (0.71–0.88)	0.75 (0.57–0.86)	0.87 (0.73–0.94)
IncArm > 30° [%]	Single	0.44 (0.28–0.59)	0.29 (0.10–0.50)	0.64 (0.40–0.82)
	Average	0.70 (0.54–0.81)	0.56 (0.25–0.75)	0.84 (0.66–0.93)
IncArm > 60° [%]	Single	0.21 (0.06–0.38)	0.13 (-0.05–0.34)	0.63 (0.37–0.82)
	Average	0.44 (0.15–0.65)	0.30 (-0.16–0.60)	0.83 (0.64–0.93)
IncArm > 90° [%]	Single	0.43 (0.27–0.58)	0.38 (0.19–0.57)	0.43 (0.15–0.70)
	Average	0.69 (0.53–0.81)	0.65 (0.41–0.80)	0.70 (0.34–0.88)
IncArm > 120° [%]	Single	0.38 (0.22–0.54)	0.32 (0.13–0.52)	0.36 (0.09–0.64)
	Average	0.65 (0.46–0.78)	0.58 (0.30–0.76)	0.63 (0.24–0.84)
IncArm > 150° [%]	Single	0.08 (-0.06–0.24)	0.05 (-0.12–0.26)	0.30 (0.03–0.60)
	Average	0.20 (-0.22–0.49)	0.13 (-0.45–0.51)	0.56 (0.08–0.82)
IncTrunk > 20° [%]	Single	0.66 (0.53–0.77)	0.57 (0.39–0.73)	0.82 (0.66–0.92)
	Average	0.85 (0.77–0.91)	0.80 (0.65–0.89)	0.93 (0.86–0.97)
IncTrunk > 30° [%]	Single	0.53 (0.38–0.66)	0.45 (0.25–0.63)	0.84 (0.69–0.93)
	Average	0.77 (0.64–0.86)	0.71 (0.50–0.84)	0.94 (0.87–0.97)
IncTrunk > 60° [%]	Single	0.20 (0.04–0.37)	0.17 (-0.02–0.38)	0.67 (0.43–0.84)
	Average	0.43 (0.12–0.64)	0.37 (-0.06–0.65)	0.86 (0.70–0.94)
IncTrunk > 90° [%]	Single	0.24 (0.08–0.41)	0.21 (0.02–0.43)	0.60 (0.35–0.79)
	Average	0.49 (0.21–0.68)	0.45 (0.06–0.69)	0.82 (0.62–0.92)
RHRmean [%]	Single	0.66 (0.45–0.80)	0.64 (0.33–0.83)	0.74 (0.52–0.88)
** **	Average	0.85 (0.71–0.92)	0.84 (0.60–0.93)	0.89 (0.77–0.96)

### Comparison of objective measurements on the first measurement day with the following days

**Fig 2:**
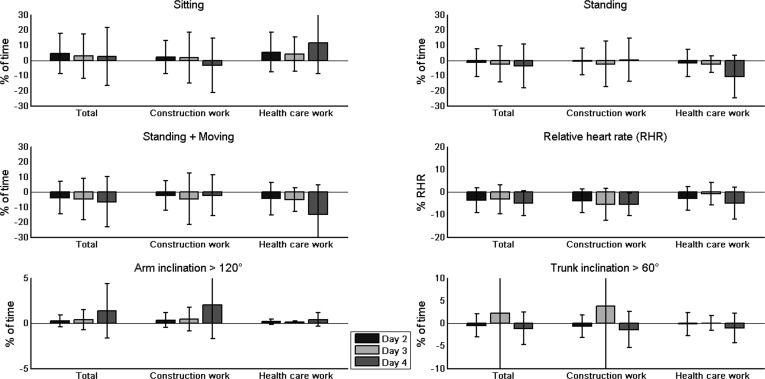
The mean of the differences between objective measurements taken over several working days and on the first day. The mean values for each variable were calculated according to individual differences between the multiday measurements and the day one measurement.

All groups were found to have spent a significantly lower amount of time with arm inclination > 120° (total: p < 0.001, construction workers: p < 0.01, health care workers: p < 0.05) on day 1 compared with the following days (see Table 5, Fig 2). For the total group and the group of construction workers, the time spent standing (p < 0.05 / p < 0.05), time spent moving (p < 0.05 / p < 0.05), trunk inclination > 60° (p < 0.05 / p < 0.05) and RHRmean (p < 0.001 / p < 0.001) were higher on day 1 compared with the following days. Furthermore, while the work hours for the total group and the group of health care workers was lowest on day 1 (p < 0.01 / p < 0.01), the group of construction workers had the lowest number of work hours on day 3 (p < 0.05).

**Table 5:** Comparison of objective measurements of several working days (Friedman Test).

	Total				Construction work				Health care work			
	N = 72				N = 42				N = 28			
	Mean ranks				Mean ranks				Mean ranks			
	Day 1	Day 2	Day 3	p-value	Day 1	Day 2	Day 3	p-value	Day 1	Day 2	Day 3	p-value
Time	1.74	2.34	1.92	**< 0.01**	1.90	2.29	1.80	**< 0.05**	1.48	2.41	2.11	**< 0.01**
Sit [%]	1.90	2.09	2.01	n.s.	1.89	2.07	2.04	n.s.	1.88	2.12	2.00	n.s.
Stand [%]	2.24	2.00	1.76	**< 0.05**	2.23	2.09	1.68	**< 0.05**	2.31	1.79	1.90	n.s.
Move [%]	2.27	1.92	1.81	**< 0.05**	2.30	1.89	1.80	**< 0.05**	2.26	1.98	1.76	n.s.
Steps [steps/h]	2.06	2.01	1.93	n.s.	2.01	1.98	2.01	n.s.	2.17	2.12	1.71	n.s.
IncArm > 30° [%]	1.90	1.86	2.25	n.s.	2.06	1.68	2.26	**< 0.05**	1.55	2.26	2.18	n.s.
IncArm > 60° [%]	1.90	1.87	2.23	n.s.	1.78	1.96	2.26	n.s.	2.13	1.74	2.13	n.s.
IncArm > 90° [%]	1.81	2.11	2.08	n.s.	1.76	2.17	2.08	n.s.	1.87	2.05	2.08	n.s.
IncArm > 120° [%]	1.58	2.25	2.18	**< 0.001**	1.58	2.35	2.08	**< 0.01**	1.55	2.11	2.34	**< 0.05**
IncArm > 150° [%]	1.82	2.13	2.05	n.s.	1.83	1.99	2.18	n.s.	1.84	2.42	1.74	n.s.
IncTrunk > 20° [%]	2.14	1.92	1.94	n.s.	2.09	1.88	2.03	n.s.	2.18	2.05	1.78	n.s.
IncTrunk > 30° [%]	2.09	1.92	1.99	n.s.	2.04	1.96	2.00	n.s.	2.23	1.80	1.98	n.s.
IncTrunk > 60° [%]	2.26	1.78	1.96	**< 0.05**	2.31	1.69	2.00	**< 0.05**	2.13	1.95	1.93	n.s.
IncTrunk > 90° [%]	1.97	1.91	2.13	n.s.	2.01	1.88	2.11	n.s.	1.83	2.00	2.18	n.s.
RHRmean [%]	2.56	1.66	1.78	**< 0.001**	2.80	1.66	1.55	**< 0.001**	2.24	1.71	2.06	n.s.

## Discussion

Knowledge of the role of workplace mechanical exposures in the pathogenesis of musculoskeletal disorders depends on the valid measurement of these exposures. The present study examined the association between exposures that were subjectively reported via questionnaires and objectively measured daily activities (sitting, standing, moving), postures of the trunk and arm, and RHR. The objective recordings were performed continuously over up to four consecutive working days. The subjective measurements were administered both at baseline prior to the first recording day and at the end of the work period on the first day of the objective measurements.

Daily activities—In the total group, analyses of the subjective and objective measurements on the first measurement day showed low correlations for time spent standing and moderate correlations for time spent sitting. The participants were not able to accurately estimate their daily activities on a working day. The lower correlations for time spent standing could be related to the participants’ interpretation of the question “How often in your daily work are you exposed to work standing?” It is possible that the participants could not discriminate between standing work and work in a moving upright position (neither still or walking). The higher correlations found for the sum of the objectively measured time spend standing and moving support this hypothesis. Depending on the study aim, the applied question should be more specified to differentiate between work when standing in one place or work in an upright position. Moderate correlations were found between subjectively measured time spent standing and the sum of the objectively measured time spent standing and time spent moving. In terms of group differences, the construction workers showed higher correlations between objectively and subjectively measured daily activities than the health care workers did.

*Postures of the arm and the trunk—*The correlations between subjectively and objectively measured arm inclination in the total group were low for arm angles > 60–> 120°. Trunk inclination > 60° showed a low correlation with subjective measures. For the construction workers, correlations ranging from 0.48 to 0.73 were found for arm inclination of > 60–> 120°, and no correlations were found for trunk inclination. The health care workers exhibited no correlations between subjective and objective measures of arm and trunk inclination. Except for arm inclination in the group of construction workers, the accuracy of subjective posture measurements was low. One reason for the low accuracy may be the way that the workers recalled a work day; they could have thought of the frequency with which they performed work tasks with specific postures. The inclinometers measure the exact angle of a body segment, and small and frequent periods with an angle outside a specific range are not detected as an exposure. Therefore, the total measured amount of the exposure duration may be lower than what the participant remembered.

*Physical exhaustion—*The questions “How physically demanding was your work today?” and “How often in your work today were you exposed to increased breathing?” were not correlated with the RHR mean. This may be explained by the absence of constant physical exposure during the working day: Frequent small breaks may lower the mean heart rate per day, despite high heart rates in situations with exposures. It is possible that the workers selectively remembered the higher-effort situations.

The differences between the groups may be partly explained by the difference in work tasks performed [16]. Construction work commonly consists of periods of repeated work tasks, e.g., building a brick wall the whole day. Health-care work consists of work cycles with more variation in movements and more tasks performed on demand. These factors may also influence the workers’ recall of exposures during a single working day.

The computed regression analyses showed the highest explained variances for the objective measurements of time spent sitting (R^2^ = 0.559) and time with hands above shoulder height (R^2^ = 0.420) on a single working day. On average, the participants overestimated the duration of exposures. The overestimation was higher for postures (e.g., sitting, β-value: 0.498) than for activities (e.g., hands above shoulder height / arm inclination > 60°, β-value: 0.063). Simplified, a self-reported time spent sitting of 50% of the working day will correspond an actual duration of approximately 25%. Similar results were found by Teschke and colleagues, who also found an overestimation of the duration of postural positions with questionnaires [15]. One should note that self-reports represent the perceived exposure, but other factors (e.g., psychosocial, psychological, physical fitness) may also influence the individuals’ judgment, leading to possible bias / overestimation. To determine the actual objective exposure from self-reports, specific models should be developed. In a recent study, Gupta and co-workers could predict 63% of the actual time the subjects were physically active or sedentary using a predictive model based on individual parameters and self-reported activities [34].

When comparing the correlations of the objective and subjective measures on day 1 and the mean values of objective measures of several days to the baseline measurements, contrasting effects can be observed. For the time spent standing and sitting and the association between the question “How physically demanding was your work today?” and the RHR mean, the correlations were higher when the objective average values were compared with the subjective baseline measurements. The correlations between arm inclination and the corresponding subjective measures where higher when the single-day measurements were analyzed. It can be assumed that the daily activities and the physical exposure would on average be constant over time in a specialized occupation, while the postures would be dependent on the actual work task, especially in the case of construction work. In longitudinal studies, these differences may be important when inquiring about exposures on single days or during a work period.

Technical recordings from a single day are representative if the variation of the mean exposure across the days is minimal [35]. Measurements performed on a single work day are useful for jobs with light and repetitive work tasks [36]. The present study found a higher degree of reliability for all of the objectively measured variables when measuring several consecutive working days compared to single day measurements. Although the reliability for the total group average measures of daily activities and RHRmean were good or excellent, the reliability of arm inclination and trunk inclination ranged from unacceptable to good, depending on the degree of inclination. In particular, for the highest amplitudes (arm inclination > 150°, trunk inclination > 60° and > 90°), the reliability was unacceptable. When comparing construction and health care workers, the main differences could be found for arm and trunk inclination. Construction workers had an unacceptable to questionable reliability for all variables of arm inclination. However, health care workers maintained an acceptable or good reliability when measuring arm inclinations of > 30°, > 60° and > 90°. Concerning trunk inclination, construction workers showed a strong decreasing reliability with an increasing inclination amplitude (good to unacceptable), whereas health care workers showed an excellent or good reliability.

This leads to the question of what causes these differences in reliability for the various groups or variables. When analyzing day-to-day differences, we found that all of the groups had shorter work periods and the lowest duration with arm inclination > 120° on the first day of measurement. Additionally, the construction workers exhibited higher values for time spent standing and moving, trunk inclination > 60° and heart rate parameters on day 1. One possible reason for these differences could be the application of the measurement equipment, which occurred during the first 30 minutes of day 1, in combination with occupation-specific work tasks. Construction workers may have had to finish the same work in less time on the first day, and their work tasks may be more dependent on the nature of the construction project or the work of other colleagues. In contrast, health care workers have a more continuous set of tasks with more frequent small breaks in between, which may compensate for lost time in the beginning of a work shift. The higher RHR found on day 1 for the construction workers supports the possibility of a higher work speed on day 1. However, the presence of an observer could also have had an impact on the participant’s heart rate. A possible consequence of all these facts might be a reduced construct validity, resulting in a decreasing reliability of the objective measurements that attempt to describe the exposure of a typical working day. Therefore, conducting measurements over several days is recommended, for both working sectors that were examined in this study.

### Methodological considerations

In this study, two sectors with unequal gender distributions were examined: construction and health care. The aim of this study was not to examine gender differences. Still, regression analyses showed no significant effect of gender on the association between objective and subjective measurements in the adjusted models. The results can be seen as representative for both sectors with their typical gender distributions. Other occupational sectors may show different results.

When comparing objective and subjective measures, errors must be taken into account depending on the precision of the questions asked and the participants’ interpretations of the questions. The questionnaire asked about the working time spent with the hands above shoulder height. Objectively considered, this question implies a wide range of the upper arm elevation (0–180 degrees, depending on individual constitution and the angle in the elbow). Arm inclination was objectively measured in a range of severities of the exposure (30, 60, 90, 120 and 150 degrees). Additionally, subjective and objective measurements examine different outcomes, such as the position of the hand and the elevation of the arm. Because of the anatomy of the body, the position of the hand depends on the inclination of the upper arm, but there are also some degrees of freedom because of the angle in the elbow and the shoulder. When examining the association of neck and shoulder pain with the risk factor “Work with elevated arms” [37], other or modified questions asking about arm elevation may achieve higher correlations to objectively measured arm inclination. In contrast with these assumptions, the subjectively (“How often during work today were you exposed to work with forward-bent trunk”) and objectively measured trunk inclination showed almost no significant associations.

The bias in the association of subjective and objective measurements could also be generated as a result of recording only the inclination of the dominant upper arm, while asking for bilateral information regarding “hands above shoulder height”. Additionally, although the inclinometers had a sample frequency of 30 Hz, the questionnaire measured the duration of the exposures in six categories ranging from 0 to 100%.

## Conclusion

The self-reported measurement tools used in this study cannot provide an accurate description of mechanical exposures neither in construction nor health care work. Self-reports showed greater precision for the measurement of daily activities, when several work days rather than single days were examined. The precision of the arm posture measurements was higher when single days were assessed. Nevertheless, objective measurements are necessary. Measurements over several work days are recommended to detect the entire exposure variance. When performing longitudinal studies, repeated objective measurements of activities, postures and cardiovascular exposures are necessary to obtain better knowledge regarding the effects of these exposures on MSD. The application of measurement equipment should not affect the participants’ work or hours worked. To adjust for overestimated exposures in questionnaires, detailed regression models are necessary and will require further investigation.

## Abbreviations

HR: Heart rate
ICC: Intraclass Correlation Coefficient
IncArm: Inclination of the arm
IncTrunk: Forward inclination of the trunk
Max: Maximum
Min: Minimum
MSD: Musculoskeletal disorders
MSI: Musculoskeletal severity index
PSI: Psychological severity index
RHR: Relative heart rate
SD: Standard deviation
